# Prevalence, determinants and perception of use of skin lightening products among female medical undergraduates in Nigeria

**DOI:** 10.1002/ski2.46

**Published:** 2021-05-20

**Authors:** O. G. Egbi, B. Kasia

**Affiliations:** ^1^ Department of Internal Medicine Niger Delta University Amassoma Bayelsa State Nigeria; ^2^ Department of Chemical Pathology, Niger Delta University Amassoma Bayelsa State Nigeria

**Keywords:** acne, cancer, cosmetics, diabetes

## Abstract

**Background:**

Skin Lightening Products (SLP) are common in Africa especially in Nigeria. Adverse effects from these products present a public health concern. Data on the use of these products among medical students is scanty.

**Objectives:**

The aim of the study was to determine the prevalence, determinants and perception of use of SLP among female undergraduate medical students in Nigeria.

**Methods:**

A cross‐sectional descriptive study was conducted among female medical students selected by convenient sampling. An on‐line survey was done with google forms using a semi‐structured self‐administered questionnaire containing participants’ biodata, history, pattern and perception of use of SLP. Data was analyzed using IBM SPSS Statistics 20.0.

**Results:**

A total of 110 respondents completed the study with over half (62; 56.4%) of them ranging between 20 and 24 years of age. The prevalence of SLP use was 45/110(40.9%) with facial cleansers being the commonest product used [23/45(51.1%)]. Over 80% of participants knew SLP could cause adverse effects with ‘skin irritation’ being the most identified [71/110(64.5%)]. Although most (80%) respondents did not think that light skin was superior to dark skin, ‘removal of discoloration/dark spots’ (40%) and ‘cosmetic reasons’ (37.8%) were the commonest reasons for use. Determinants of use of SLP were light skin color (OR 3.8, 1.572–9.318), history of use among relatives (OR 3.3, 1.384–7.793) and awareness of adverse effects (OR 3.3, 1.129–9.740).

**Conclusion:**

The prevalence of use of SLP was quite high among the respondents and was predicted by ‘skin color’, ‘use among relatives’ and ‘awareness of adverse effects’. Education of the general public on their adverse effects is paramount to avoid harmful use.

## Graphical abstract

Factors influencing type of skin lightening products purchased. NAFDAC, National Agency for Food and Drug Administration and Control

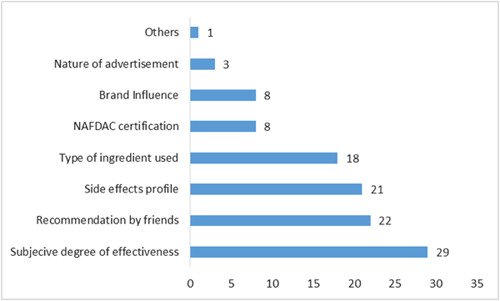

1

**What is already known about this topic?**

- The prevalence of skin lightening in general population of Nigeria is very high.
- Use of SLP is also prevalent in other parts of Africa, Asia and other parts of the World.
- The prevalence of skin lightening among Nigerian female undergraduates is similarly high.
- Use of SLP is higher among dark skinned females, and more commonly influenced by wrong perception about skin color and SLP.

**What does this study add?**

- The prevalence of skin lightening is also high among medical students though lower than what obtains in the general population.
- The awareness of adverse effects of SLP was high among the female medical students.
- Skin color was a determinant of skin lightening with lighter skinned individuals having increased odds for use of SLP compared with the dark skinned.
- Other determinants of use of SLP were history of use among relatives and awareness of adverse effects.

## 2 INTRODUCTION

Skin lightening creams (also known as skin bleaching, whitening, brightness or fading creams) contain products such as corticosteroids, hydroquinone, mercury salts, and a variety of other substances which work by reducing melanin to attain a lighter skin color. The motivation for the use of these products may rarely be to treat pigmentary disorders like melasma but more often they are used for cosmetic reasons.^1^

Numerous cutaneous side effects including atrophy, striae, telangiectasia, acne vulgaris, allergic and irritant contact dermatitis, steroid rosacea, hirsutism, infection and ochronosis have been reported with skin lightening products (SLP).^2^, ^3^, ^4^, ^5^ Other adverse effects may include systemic complications like Cushing's syndrome, diabetes mellitus, hypertension, organ failure involving the kidneys and liver, cancers and ocular complications such as cataract and glaucoma.^2^, ^3^, ^4^, ^5^

The cosmetic use of SLP is common in Africa, Asia and many other parts of the world. It is believed that the effect of colonialism in some of these countries and the belief in white supremacy may have fueled the use of SLP in Asia and Africa as far back as historic times^6^ A recent meta‐analysis global pooled lifetime data on use of SLP reported a prevalence of 27.7% and an estimated rate of 27.1% among Africans.^7^ Reported prevalence of skin toning among female university students in Ghana was 40.9%.^8^ Nigeria has about the world's highest percentage of women engaged in skin lightening. According to the World Health Organization, 77% of women in Nigeria use skin lightening products.^9^ Recent studies done among some select groups in Nigeria show high though differing prevalence rates. For instance, while 77.3% of traders in Lagos State practiced skin lightening^10^ 48.1% of university undergraduates in Maiduguri used SLP.^11^

This study, however, focused on medical undergraduates. We hypothesize that the use of SLP among medical students would be low. This is because it is expected that their knowledge of adverse effects of the products may serve as a deterrent from abuse. Medical undergraduates are a potentially influential group of people among their peers in the society and are often looked upon as role models. As potential physicians, they could assist in helping patients develop healthy lifestyles. It is, therefore, imperative that they have the right belief and practice in health‐related issues. A study of the perception and practices concerning their use of SLP will be paramount in addressing this problem in the society. This may determine the type of influence they will wield as regards the use of the products. This study was, therefore, aimed at determining the prevalence, pattern and perception of use of SLP among female medical undergraduates of a Nigerian University. We studied only females here because women are known to engage in skin lightening practices much more often than men do. Reasons for use of SLP among women may include perceived societal association of white skin with beauty, higher status and privilege.^1^, ^12^, ^13^ This study may provide the requisite knowledge and impetus needed for government and other relevant stakeholders in making policies aimed at curbing the harmful practice of indiscriminate skin lightening.

## 3 METHODS

### 3.1 Study setting

The study was carried out among female medical students of the Niger Delta University (NDU) located in Wilberforce Island in Bayelsa State. The oil‐rich Bayelsa State is a prominent state in the Niger Delta Region of Nigeria and lies within the south‐south geo‐political zone of the country. With a population of just under 2 million and total area exceeding 8 000 square miles, the state currently hosts three universities. However, NDU is the only one with a full‐fledged medical school at the moment.

### 3.2 Study population and design

This was a cross‐sectional descriptive study done among female medical undergraduates from first to 6th year of study. For the purpose of this study, participants were classified into three classes based on their educational levels. Those in the first two years of study comprised ‘the lower educational class level’ while ‘the middle educational class level’ was made up of those in the third and fourth year of study. The higher classes on the other hand, comprised those in the fifth and sixth year. The students belong to varying socio‐economic classes. They all pay a yearly tuition fee with the exception of those on scholarship. Only those that completed the questionnaire were admitted for the study. Those that were unwilling or who did not give a written informed consent were excluded from the study.

### 3.3 Study procedure

The study employed the use of a pre‐tested on‐line survey containing self‐administered semi‐structured questions adapted from previous studies^14^ and developed on google forms by an expert web designer. The questionnaire were sent to three Consultant Dermatologists for content review and subsequently modified in line with their input. The questionnaire in the final form alongside the informed consent form was sent to the class representatives at the various levels who in turn sent them to their classmates via their class WhatsApp platforms. The platforms were comprised of all members of each class, coordinated by the class representatives and have previously been used to disseminate important information to members. The questions were mainly close‐ended but also had a few short‐phrased open ended questions where certain clarifications were needed. Some of the questions had room for multiple responses. The questionnaire was divided into several sections. Section A contained questions on demographic and social data such as age group, state of origin, religion, self‐report of skin color (whether dark or light skinned), use of skin lightening lotions and reasons for use. Section B contained information on knowledge and respondents perception of light skin and skin lightening as well as sources of information while section C inquired about details of the SLP and how it was being used. Section D, on the other hand inquired about how the products were sourced and the level of satisfaction of users. Sections E and F centered on questions bordering on knowledge of side effects and complications of SLP, respectively. Respondents were expected to submit their responses to a general on‐line pool of responses by clicking on a button after answering the questions. The responses were automatically generated and saved in the forms. Although the survey was posted on the general class WhatsApp page, the convenient sampling technique was the method used as only students who had internet access during the course of the study and who were willing to spare their data, participated in the survey. The survey was carried out during a compulsory student break occasioned by the strike action of the Academic Staff of Universities (ASUU). It was not possible to physically administer questionnaire to the students during this period. The entire study from conceptualization and design to questionnaire administration, data analysis and the initial draft of the manuscript took a duration of 5 months from September 2020 to January 2021.

### 3.4 Ethical consideration

Ethical clearance was sought and obtained from the Research Ethics Committee (REC) of the Niger Delta University Teaching Hospital (NDUTH) with ethical clearance no: NDUTH/REC/2020/026/0198. Individual participant consent to participate in the study was given by signing the accompanying informed consent form.

### 3.5 Statistical analysis

The saved data was imported into excel and then eventually into IBM SPSS 20.0. Categorical variables were expressed in form of proportions and frequencies. The prevalence of use of SLP was defined as the proportion of those using SLP divided by the population studied. The characteristics of users of SLP were compared with those of non‐users using chi‐square. Variables associated with SLP was determined using univariate analysis and those found to be significant (with *p* < 0.1) were entered into the multivariate model using backward selection approach. For the final analysis, the 95% confidence limit was used with a *p* < 0.05 considered significant for a two‐tailed test.

## 4 RESULTS

### 4.1 Socio‐demographic data of respondents

A total of 110 participants completed the study out of 116 students who began the survey. Most of the participants (62; 56.4%) were in the age bracket 20–24 years. About 27 (24.5%) of them were below 19 years old while only two (1.8%) were older than 35 years.

A total of 72 (65.5%) participants were indigenes of Bayelsa State in Nigeria while the others were from other states. Majority, 107 (97.3%) were Christians, 2(1.8%) were Muslims while 1(0.9%) had no religion. Up to 69 (62.7%) of the participants were in the middle educational class levels. About 63 (57.3%) of the participants had dark skin color while 47 (42.7%) had light skin. About 48 (43.6%) participants had a close relative that used SLP. The characteristics of the respondents is detailed in Table 1.

**TABLE 1:** Characteristics of participants using SLP compared with those not using SLP

Characteristic	Total participants	Use of SLP	Use of SLP
	*n* (%)	Yes *n* (%)	No *n* (%)
Age (yrs)			
15–19	27(24.5)	12(26.7)	15(23.1)
20–24	62(56.4)	23(51.1)	39(60.0)
>25	21(19.1)	10(22.2)	11(16.9)
*p* = 0.637			
Class			
Lower	17(15.5)	8(17.7)	9(13.8)
Middle	69(62.7)	26(57.8)	43(66.2)
Higher	24(21.8)	11(24.5)	13(20.0)
*p* = 0.669			
State of origin			
Within Bayelsa	72(65.5)	32(71.1)	40(61.5)
Outside Bayelsa	38(34.5)	13(28.9)	25(38.5)
*p* = 0.299			
Use of SLP among relatives			
Yes	48(43.6)	25(55.6)	23(35.4)
No	62(56.4)	20(44.4)	42(64.6)
*p* = 0.036			
Skin color			
Dark	63(57.3)	20(44.4)	43(66.2)
Light	47(42.7)	25(55.6)	22(33.8)
*p* = 0.024			
Perception of skin color			
Right	30(27.3)	12(26.7)	18(27.7)
Wrong	80(72.7)	33(73.3)	47(72.3)
*p* = 0.905			
Knowledge of ingredients			
Yes	58(52.7)	25(55.6.)	33(50.8)
No	52(47.3)	20(44.4)	32(49.2)
*p* = 0.621			
Awareness of side effects			
Yes	91(82.7)	34(75.6)	57(87.7)
No	19(17.3)	11(24.4)	8(12.3)
*p* = 0.098			

Abbreviation: SLP, skin lightening products.

### 4.2 Prevalence and characteristics of users of SLP

A total of 45 participants used SLP, giving a prevalence of 40.9%. The characteristics of users of SLP are also compared with those of non‐users in Table 1. Individuals who used SLP were more likely to be light skinned (*p* = 0.034) with a positive history of use of SLP in a close relative (*p* = 0.036). While 31.7% light skinned ladies used SLP, 53.2% light skinned ladies used SLP. Similarly while 52.1% of those with a history of use of SLP among close relatives used SLP, only 32.3% of those without a similar history used SLP (Table 1).

### 4.3 Knowledge and perception of SLP

About 91 (82.7%) knew that there were adverse effects associated with use of SLP, five (4.5%) did not know there were adverse effects while 14(12.7%) were not certain. The identified side effects (with allowance for multiple options) included skin irritation (71 responses), discoloration (60 responses), skin irritation (60), sunburn (34), skin veins (34), skin peeling (28) and acne (27 responses). Others were rashes (20), atrophy (14), infections (8), ochronosis (6) and stretchmarks (2 responses).

In terms of responses given on complications of SLP, only 24 (21.9%) respondents knew that kidney disease was a possible complication. While 17 (15.5%) thought kidney complication was not a possibility, 31 (28.2%) were not certain. About 17 (15.5%) participants correctly identified liver failure as a possible complication. While 18 (16.4%) thought liver failure could not occur with SLP, 38 (34.5%) respondents were uncertain. ‘Cancer’ was identified as a possible complication of SLP in 71 (64.5%) respondents while 10 (9.1%) were uncertain. About 23 (20.9%) respondents also correctly identified fetal toxicity as a complication of SLP while eight (7.3%) believed it was not but 37(33.6%) respondents were uncertain. Only one (0.9) respondent correctly identified diabetes mellitus as a possible complication of SLP. While 31(28.2%) did not think diabetes was a possible complication, 33 (30%) respondents answered that they were not certain.

About 55 (50.0%) respondents claimed that they knew the ingredients in SLP while 51 (46.4%) did not. Four (3.6%) participants gave no response. The harmful ingredient implicated by respondents were hydroquinone in 47(42.7%), mercury 29(26.4%), arsenic 11(10.0%), steroid 9(8.2%), chromium 4(3.6%), and cadmium 3(2.7%) responses. The source of information about SLP among users were internet and social media in 81(73.6%). friends (22; 20.0%). books/newspaper/magazine (20; 18.2%) TV/radio (14; 12.7%).

About 23 respondents (20.9%) perceived that men considered women with lighter skin to be more beautiful. Five (4.5%) responses indicated a perception that light skin was more beautiful and healthier while the perception that light skin helps in getting better jobs was reported by only three (2.7%) participants. Also, only two (1.8%) respondents felt light skin implied belonging to a higher social class while one (0.9%) respondent opined that light skin increases chances of getting married. Eighty (72.7%) respondents, however, did not think light skin was particularly superior or advantageous over dark skin.

### 4.4 Practice and use of SLP among the respondents

The frequency of use of the SLP by the respondents is shown in Table 2. The reasons for use of SLP was purely for cosmetic reasons in 17 (15.5%) while it was intended to treat discoloration or dark spots in 18 (16.4%) and acne in 10 (9.1%), without a physician's prescription.

**TABLE 2:** Skin lightening practices among users of SLP

Variable	Frequency (%)
Prevalence (*n* = 110)	45 (40.9)
Type of product (multiple responses allowed)	
Facial cleanser	23(51.1)
Facial toner	21(46.7)
Sunblock/serum	18(40.0)
Facial moisturizer	17(37.8)
Anti‐age	2(4.4)
Others	14(31.1)
Make of product (*n* = 45)	
Local	14(31.1)
International	31(68.9)
Frequency of use (*n* = 45)	
In the past	5(11.1)
Rarely	12(26.7)
Occasionally	11(24.4)
Sometimes	11(24.4)
Always	6(13.4)
Reasons for use (*n* = 45)	
Cosmetics	17(37.8)
Discoloration or dark spots	18(40.0)
Acne	10(22.2)
Satisfaction with use (*n* = 45)	
Yes	36 (80.0)
No	9 (20.0)

Out of all that used SLP, 27 (60.0%) respondents applied it on the face while 16(35.6%) respondents used it everywhere. Upper part and lower part application was found in only one (2.2%) respondent each. Of the 45 that used lighteners, 23 (51.1%) used facial cleanser while 21 (46.7%) used facial toner. Over 50% of users of SLP got the products from the supermarket. About 10 (22.2%) respondents got the products from specialty beauty shops while eight (17.8%) purchased products on‐line. Other sources were ‘drug stores’ in three (6.7%) participants and self‐formulation in one (2.2%) (see Table 2). About 31 (68.9%) users of SLP usually checked for names of ingredients in the products before purchase.

Choice to purchase particular SLP among users was mainly influenced by the extent of the subjective feeling of degree of effectiveness of the product by the participants' in 29(64.4%) and recommendation by friends in 22(48.9%). Other factors influencing purchase included side effect profile, type of ingredients used and certification by the National Agency for Food and Drug Administration and Control (NAFDAC); which is the major regulatory body for pharmaceuticals and related products in Nigeria.

Univariate logistic regression identified the following factors in association with use of SLP: light skin color (OR = 2.81, 0.159–0.785 *P* = 0.012), use of SLP among relatives (OR – 2.65., 1.185–5.902; *P* = 0.018) and awareness of side effects of products (OR = 2.38, 0.872–6.475 *p* < 0.09). On multivariate analysis, all three factors were found to be independent predictors of use of SLP. Compared with dark skinned individuals, light skinned participants had almost 4 times increased odds for use of SLP (AOR = 3.82; 1.572–9.318). Also, participants that had a close relative using SLP had about three times higher odd for use of SLP more than those who did not have relatives that used SLP (AOR = 3.32; 1.384–7.793). Similarly, participants that were not aware of the adverse effects of SLP also had over three times increased odd for use of SLP (AOR = 3.32; 1.129–9.740 [Table 3]).

**TABLE 3:** Factors associated with use of skin lightening products

Factor	Univariate analysis			Multivariate analysis		
	OR	95% CI	*P*	OR	95% CI	*P*
Age (yrs)						
15–19	1		0.716	‐	‐	‐
20–24	1.117	0.347–3.594	0.853			
≥25	0.774	0.278–2.166	0.625			
Class level						
Lower	1		0.754	‐	‐	‐
Middle	1.429	0.551–3.707	0.463			
Upper	1.091	0.358–3.322	0.878			
State of origin						
Outside Bayelsa	1	0.670–3.641	0.302	‐	‐	‐
Bayelsa	1.562					
History of use of SLP among relatives						
Yes	2.645	1.185–5.902	0.018*	3.322	1.384–7.793	0.007*
No	1					
Skin color						
Dark	1		0.012*	3.827	1.572–9.318	0.003*
Light	2.815	0.159–0.785				
Perception of skin color						
Right	1					
Wrong	1.137	0.469–2.757	0.776	‐	‐	‐
Knowledge of ingredients						
Yes	1.479	0.671–3.257	0.322	‐	‐	‐
No	1					
Awareness of side effects						
Yes	2.375	0.872–6.475	0.091	3.316	1.129–9.740	0.029*
No	1					

Abbreviation: SLP, Skin Lightening Products.

## 5 DISCUSSION

This is one of the few reports focused on use of SLP among medical students in Nigeria. Although the use of SLP has been reported to be high among university undergraduates in general, there have only been scanty reports on medical students who are expected to be more knowledgeable with the right health, related attitude. The study reported the prevalence of skin lightening among female undergraduate medical students of the Niger Delta University as 40.9%. This is higher than the global and African prevalence reported by Sagoe et al.^7^ (27.7% and 27.1%) respectively, exactly same as that reported in Ghana (40.9%)^8^ but lower than the prevalence of 64.9% among Nigerian undergraduates of University of Benin^15^ and the 77.3% among Nigerian traders.^10^ This is also lower than the prevalence rate of 52.2% obtained in a study by Yusuf et al among health science students in Somaliland.^16^

The high use of facial toners, cleansers and moisturizers among participants in this study, is in keeping with the belief of promoting skin/facial beauty, attracting the opposite sex and increasing female confidences.^12^, ^13^ This is, however, disturbing as these products have also been shown to contain harmful ingredients like mercury and hydroquinone. Although the analytes in these products reveal levels of mercury and hydroquinone often less than the threshold values, they nonetheless pose significant health risks, as with continuous use, the ingredients tend to accumulate in the kidneys and liver with subsequent damage.^16^ The higher percentage of international products used among participants is reflective of the poorly regulated importation and access of products into developing countries of Africa.^17^ Although majority of respondents were aware of the side effects of the products, quite a number of them still patronized them. For this group of individuals, perceived benefit of the products probably outweighed the risk. Similarly, according to report by Amodu et al, despite good awareness of adverse effects of SLP among female undergraduates in Northern Nigeria, the products were still commonly used for beautification.^11^ Hydroquinone was the most common harmful ingredients of SLP identified by the respondents. This is also corroborated by the Somaliland study, where participants mentioned hydroquinone as the highest‐ranking toxic ingredients in the products.^18^ Hydroquinone is notorious in the literature for its harmful effect as one of the primary active ingredients in many SLP.^19^

It is also noteworthy, that a higher percentage of respondents in this study did not think that lighter skin was particularly more superior or advantageous over dark skin. The major reason for use of SLP was for removal of dark or discolored spots. A few others believed it would make their skin healthier or more beautiful. It is quite interesting, however, that light skin color was found to be a determinant of SLP use in this study. Those who were light skinned had a higher tendency to use these products compared with dark skinned respondents. One would have expected the converse to be true. However, anecdotal evidence seems to suggest that light skinned individuals commonly use these products presumably to preserve their skin color or make it brighter. It is possible that the light skinned women use these products to counteract the skin tanning effects of the sun (to which they are more susceptible compared with dark skinned women) in tropical areas. Even white women have used SLP to maintain radiant skin devoid of hyperpigmentation as a result of being exposed to heat or maturation process.^20^ It is also possible that the trend is changing with dark skinned individuals now wishing to preserve their color.

Other determinants of use of SLP in this study were ‘use of SLP among relatives’ and ‘awareness of side effects’. Close relatives of individuals using SLP in our study were more likely to use the products themselves. Similarly, a Sudanese study reported that the odds of using skin whitening products in female undergraduates who had mothers or sisters bleaching were 7.8 times higher and two times higher in females who had other relatives bleaching compared with women who had no relative bleaching.^21^ This finding is further corroborated in the Somalian study, where almost half of respondents who practiced skin lightening alluded to the influence of family members and friends.^18^ A good proportion of participants in this study made a choice of SLP based on recommendation of friends. The influence of peer pressure must therefore also be considered and further explored in this age group.

Awareness of adverse effects of SLP was another potent determinant of use. Respondents who had no awareness of adverse effects were also about 3.3 times more likely to use the products compared with those who were aware of their side effects. This creates a window of opportunity for possible intervention to help reduce the use of SLP and mitigate the attendant complications. Although majority of our respondents were aware of the cutaneous effects of the products, a larger proportion were unaware of their extra‐cutaneous and long‐term complications including the systemic effects. More awareness needs to be created about these complications for medical students and indeed for all and sundry. This can be done for students by including it in their curricula at all levels. Additionally, other stakeholders such as pharmacists and owners of supermarkets, drug stores, specialty beauty shops, on‐line marketers and self‐formulators of products need to be specially targeted for education and counseling on adverse effects of SLP and possible alternatives. This is important as these facilities were the major gateways for access to these products. The media should be used to create awareness about the dangers of skin bleaching. The internet and social media should be specially targeted as this was a major source of information about SLP in our study. Advertisement that promotes fair skin as a symbol of beauty and use of fair‐skinned models to promote cosmetics targeting the black market should be discouraged. The old mantra regarding black as being beautiful should be regarded. All relevant government agencies should take concrete steps to restrict access to SLP that contain potentially harmful products. Heavy penalties should be placed on companies that continue to manufacture these products and market them to third world countries.

A limitation of the study is the small sample size obtained from only one institution and the non‐probability sampling technique used. The design of the study also makes it prone to response and recall bias. Despite these limitations, however, the study is suggestive of a high prevalence of use of SLP among medical undergraduates though lower than the overall rate in Nigeria. Majority of the respondents in this study have good knowledge of adverse effects of SLP though less knowledgeable about their long‐term complications and systemic effects. The use of SLP appears to be determined by awareness of the side effects, skin color or history of use of products among close relatives. Education and advocacy is needed to avoid the use of harmful products.

## CONFLICT OF INTERESTS

No conflict of interests have been declared.

## Data Availability

The data that support the findings of this study are available from the corresponding author upon reasonable request.
